# On the Nonspecific Resistance in Burn Injury: Pathophysiological Aspects (Review)

**DOI:** 10.17691/stm2020.12.3.11

**Published:** 2020-06-28

**Authors:** A.V. Pereshein, S.V. Kuznetsova, O.N. Shevantaeva

**Affiliations:** Assistant, Department of Pathological Physiology; Privolzhsky Research Medical University, 10/1 Minin and Pozharsky Square, Nizhny Novgorod, 603005, Russia; Associate Professor, Department of Pathological Physiology; Privolzhsky Research Medical University, 10/1 Minin and Pozharsky Square, Nizhny Novgorod, 603005, Russia; Professor, Department of Pathological Physiology Privolzhsky Research Medical University, 10/1 Minin and Pozharsky Square, Nizhny Novgorod, 603005, Russia

**Keywords:** nonspecific resistance, burn injury, burn sepsis, neutrophils, monocytes, macrophages.

## Abstract

An analysis of nonspecific resistance in burn patients is conducted. The role of subpopulations of neutrophils and monocytes/ macrophages in severe burn injury is discussed. The significance of blood cells for the burn-induced immune dysfunction, susceptibility to sepsis and multiple organ failure is underscored. The involvement of secondary complications in the development of morbidity and mortality in patients with burn injury is shown. New approaches to identifying individuals with a risk of adverse outcome are considered.

## Introduction

The annual frequency of severe burns, according to a European study [1], ranges from 0.2 to 2.9 cases per 10 thousand population. In Russia, about 400 thousand burns are registered annually [2]. Burns cause morbidity and mortality: they account for more than 300,000 deaths per year in the world [3–6]. Treatment of burns is quite expensive as it requires prolonged hospitalization and rehabilitation [7, 8].

The main cause of deaths in hospitalized patients with severe burn injury is sepsis [8–10], which is associated with high (up to 85%) mortality [11]. Burn injury alters the skin integrity and thus violates the major anti-pathogen barrier in the body, which increases the risk of infections. In addition, in burned patients, the subsequent systemic inflammatory response is accompanied by multiple organ dysfunction and immunosuppression phase, which increases the susceptibility to nosocomial infection [12]. Often, a systemic inflammatory response syndrome masks the onset of burn sepsis, which delays the diagnosis of concomitant septicemia [13]. This factor adversely affects the outcome.

This critical effect of infection on treatment outcomes, as well as the diagnostic difficulties encountered in seriously burned patients, requires new methods of identification and characterization of these life-threatening conditions. In this context, nonspecific resistance biomarkers are needed to help improve the prognosis for patients with severe burn injury.

## Neutrophils

Polymorphonuclear leukocytes, which include polymorphonuclear neutrophils (PMN), are the key cells of the innate immune system involved in the inflammatory response, and the first ones to rush into the infected and/or damaged tissues [14]. In healthy people, approximately 100 billion neutrophils replenish and leave the circulating blood every day [15, 16]. They constitute the dominant leukocyte population in the circulating blood; they mediate the earliest immune responses to infection, and also capture and destroy invading microorganisms through phagocytosis and intracellular degradation [17]. Until recently, these functions were considered unique to neutrophils. However, ongoing research in several areas of cell biology shows that PMNs have a diverse repertoire of functional responses that go beyond simply killing microorganisms. Currently, it is recognized that neutrophils are transcriptionally active complex cells [11, 17] that produce cytokines [18], modulate the activity of neighboring cells, help resolve inflammation [19], and mobilize macrophages for the long-term immune responses [20]. Under these conditions, neutrophils with their powerful antimicrobial functions are, on the one hand, important host defenders and, on the other, a dangerous source of inflammatory mediators that damage tissues under conditions of uncontrolled inflammation [21–23].

The generation of neutrophils from hematopoietic progenitors in the bone marrow is strictly controlled [24]. The main regulator of granulocytopoiesis is the granulocyte colony-stimulating factor (G-CSF), which promotes the fixation of myeloid line progenitor cells, reduces their maturation time, and stimulates the proliferation of granulocyte precursors and the release of mature cells from bone marrow [25]. Additional signals stimulating the production and release of neutrophils can come from IL-6, IL-3, and granulocyte-macrophage colony-stimulating factor (GM-CSF) [26].

The number of neutrophils in the circulating blood is regulated by the CXCL12/CXCR4 axis (chemokine ligand 12/chemokine receptor 4) [24, 27]. Bone marrow stromal cells express CXCL12, the ligand for CXCR4, presumably bound to neutrophils and retaining them in the bone marrow [28]. Although there is not enough direct evidence of CXCR4 expression on human neutrophils in the bone marrow, plerixafor, an antagonist of the CXCR4 receptor, is able to mobilize neutrophils in the blood [29]. It was shown that co-administration of G-CSF with the CXCR4 antagonist led to a synergistic release of neutrophils [30]. The addition of G-CSF reduces the formation of CXCL12, which correlates with an increase in neutrophil mobilization [31]. In sepsis, lipopolysaccharide (LPS) and inflammatory cytokines, such as TNF-α, IL-1β, IL-6, and IL-17, can regulate the level of G-CSF [32]. Two randomized clinical trials on recombinant G-CSF were conducted; the agent was shown to increase the number and stimulate the function of neutrophils in patients with sepsis [33, 34]. The use of G-CSF is very effective in preventing septic complications in individuals with an abnormally low number of neutrophils; notably, the number of neutrophils in sepsis tends to increase [22]. Researchers have suggested that the administration of G-CSF can improve the bactericidal function of neutrophils. However, although the number of PMN cells in these patients increased, there was no increase in overall survival. These two clinical studies suggest that administration of G-CSF is only useful in patients with neutropenia.

Guerin et al. [35] found that the development of sepsis is associated with an increase in the number of immature forms of neutrophils, which was of high prognostic value 48 h after hospital admission. It is important to note that determining the number of young forms of neutrophils makes it possible to distinguish between patients with SIRS (systemic inflammatory response syndrome) and those with sepsis with a sensitivity of 89.2% and a specificity of 76.4% [36]. These data are consistent with the results of Hampson et al. [37], who showed that within 24 h after a burn, the number of circulating immature neutrophils was significantly higher than that in healthy volunteers. Their number returned to normal on day 3 after the injury, and on day 7, it increased again and remained elevated for 28 days. In addition, there was a change in the functional activity of granulocytes on days 3 and 7. The change was expressed as a decreased ability to generate an oxidative burst and a decrease in the phagocytic index, which might underlie the increased susceptibility to infection after thermal damage [38].

Thus, counting the number of immature neutrophils helps to accurately distinguish between septic and nonseptic individuals with SIRS. This is especially important in patients with burns, where sepsis is difficult to diagnose, since many of the diagnostic criteria are masked by the developing SIRS, which is typical of patients with burns >15% of the body surface area.

Normally, mature neutrophils circulate in the blood for no more than 6–10 h, and then move to tissues [14, 23, 28]. They quickly respond to inflammatory signals after tissue damage or infection and migrate to the inflamed/ damaged zone [14].

The initial period of thermal injury is characterized by neutrophil hyperactivity. A large number of bactericidal reactive products resulted from the NADPH oxidase, myeloperoxidase (MPO), or nitric oxide synthase (NOS) reactions are released from neutrophils [39].

Along with the generation of oxygen radicals, the cytotoxicity of neutrophils is mediated by granule secretion. Primary neutrophil granules (azurophilic) contain MPO and a number of neutrophil serine protease (NSP): cathepsin G (CG), neutrophil elastase (NE), proteinase 3 (PR3), and the recently discovered neutrophil serine protease 4 (NSP4) [40]. NSPs are critical for the effective functioning of neutrophils and contribute significantly to immune defense against bacterial infections [41].

The following NSP features are known:

1. NSPs can directly kill bacterial cells. It has been shown that NE destroys gram-negative *E. coli* by cleaving protein A of the outer membrane, and leading to cell death. The coordinated actions of NE, CG, and PR3 *in vivo* can neutralize *S. pneumoniae* in a phagocytic vacuole.
2. NSPs are able to cleave host proteins to produce antimicrobial peptides. The best known example is the ability of PR3 to cleave cathelicidin hCAP-18 to produce the LL-37 antimicrobial peptide. Cathelicidins are inactive when contained in specific granules. During degranulation of azurophilic and specific granules, PR3 cleaves the C-terminal part from cathelicidin, releasing the cationic bactericidal peptide LL-37 with its bactericidal activity against both gram-positive and gram-negative bacteria.
3. NSPs can attenuate bacterial virulence by inactivating some factors of the pathogenesis. *Shigella flexneri* mobile IcsA and IpaA-C proteins can be inactivated by NE, which prevents the bacteria spread in the neutrophil cytoplasm. Likewise, CG can cleave the adhesive binding factor A of *S. aureus* and remove its active domain [15]. In addition, the neutrophil azurophilic granules contain a bactericidal protein that increases the permeability of bacterial cells [42]. This protein has three types of antibacterial action: direct antimicrobial activity, neutralization of endotoxin through a direct LPS binding, and opsonic activity.

The specific granules get formed after azurophilic granules. They mainly contain a wide range of antimicrobial compounds, including calprotectin, lactoferrin, lipocalin bound to neutrophilic gelatinase (NGAL), hCAP-18, and lysozyme. Calprotectin, also called S100A8/A9, is a critical factor in the innate immune response to infection and, as shown [43], inhibits the growth of microorganisms by chelating the nutrients necessary for microbes to progress *in vivo* the ions of Fe^2+^, Mn^2+^, and Zn^2+^, which leads to the reprogramming of the bacterial transcriptome. Lactoferrin, also called lactotransferrin, is an iron-binding glycoprotein present in most human biological fluids [44, 45].

Tertiary (gelatinase) granules are both MPO- and lactoferrin-negative. They represent one of the final populations of granules formed during the maturation of neutrophils. Gelatinase granules contain several antimicrobial compounds and also store a number of metalloproteases, such as gelatinase and leukolysin.

Currently, antimicrobial peptides (AMPs) are becoming the focus of developing new strategies for treating bacterial infections [46, 47]. It is suggested that AMPs may be promising candidates for the treatment of the so-called ESKAPE pathogens (*Enterococcus faecium*, *Staphylococcus aureus*, *Klebsiella pneumoniae*, *Acinetobacter baumannii*, *Pseudomonas aeruginosa*, and *Enterobacter*), which are the group of the most “rebellious” bacteria resistant to almost all antibiotics; these pathogens are the leading causes of hospital infections, including that in burn patients [48–50].

Highly reactive oxygen species and AMPs are crucial for the effective neutrophil performance and the maintenance of phagocytosis. In addition, another concept of antimicrobial killing based on neutrophil extracellular traps (NET) has been described [51, 52]. The main components of NET are DNA, granular neutrophil proteins, and histones H1, H2A, H2B, H3, and H4 [53, 54]. Neutrophilic traps are formed in response to pro-inflammatory stimuli, of which IL-8, TNF-α, and LPS are the most significant [55]. During the formation of NET, neutrophils die, and this process is commonly called NETosis.

The cell-free DNA (cfDNA) and modified histones involved in NET were proposed to potentially serve biomarkers of sepsis [54, 56]. For example, Hampson et al. [37] showed that the plasma levels of cfDNA after thermal damage were significantly higher in those patients who developed sepsis. In addition, plasma cfDNA levels measured on the day of the injury differed between septic and nonseptic patients. Thus, the highest AUROC value was 0.935 in a multi-parameter model, which combined the phagocytic index and the number of immature granulocytes in the blood. It is important to note that circulating cfDNA is nonspecific for NETosis and can originate from apoptotic or necrotic cells, as well as from bacteria [57]. In order to provide convincing evidence of NETosis *in vivo*, researchers [37] analyzed samples of the blood plasma for the presence of citrullinated histone H3 (Cit H3). High levels of Cit H3 correlated with high levels of cfDNA, demonstrating that NETosis did occur during septic episodes and it contributed to the increase in plasma cfDNA. These data are consistent with the work of Hirose et al. [57], which showed the presence of Cit H3 only in infected patients.

Thus, the inclusion of cfDNA and Cit H3 in the sepsis risk stratification systems may be useful for clinical decision-making or for studying sepsis in patients with thermal injury.

Neutrophil-mediated cytotoxicity is implicated in damage to microcirculation vessels [58] and multiple organ injuries caused by extensive traumas, burns, and sepsis [30]. During sepsis, bacterial products and proinflammatory cytokines, such as TNF-α and IL-1β, reduce the expression of L-selectin on the surface of neutrophils and stimulate the expression of β-integrins that interact with the intercellular adhesion molecule 1 (ICAM-1) and the vascular cell adhesion molecule 1 (VCAM-1) on the vascular endothelium and thereby contribute to the high affinity adhesion to the endothelium [59]. As a result, neutrophils show a decrease in marginalization and rolling as well as reduced deformability and sequestration in the vascular region. At the same time, the neutrophil membrane becomes more rigid and less deformable — in proportion to sepsis severity [30, 60]. Sequestration of neutrophils in capillaries leads to vascular occlusion and promotes tissue ischemia and organ dysfunction, especially in the highly vascularized lungs and liver [32, 60].

Normally, the destructive effects of neutrophils in the tissue are limited by apoptosis of neutrophils. However, this process is delayed by an injury (3–5 days instead of 7–9 h) [61]. Delayed apoptosis leads to the accumulation of neutrophils, an increased release of their cytotoxic products, and the development of local tissue damage [62].

An important role in the development of multiple organ failure and impaired microcirculation is played by the interaction of neutrophils and platelets [60]. It is well known that activated platelets adhere to neutrophils by rapid surface expression of granular P-selectin protein that binds to the high affinity PSGL-1 ligand expressed in neutrophils [63]. This interaction causes further activation of the neutrophil β2-integrins LFA-1 (αLβ2) and Mac-1 (αMβ2), resulting in a massive migration of neutrophils toward distal organs [64]. It was found that the interaction of platelets and neutrophils led to a rapid release of NET [54, 65], promoting the adhesion of platelets and red blood cells, and stimulating the formation of blood clots [66]. Additionally, platelets interact with neutrophils during sepsis by triggering the TREM receptor expressed on myeloid cells in the presence of LPS; the process stimulates the neutrophil-mediated production of reactive oxygen species (ROS) and the secretion of IL-8 [67].

Numerous studies have shown that sepsis in burn patients represents a serious violation of the immune response to infection; it leads to neutrophil dysfunction and inhibits their migration capacity. Until recently, the technology used to measure neutrophil migration was limited, time-consuming, and required a large volume of blood. In 2010, Butler et al. [68] described a new microfluidic device that allowed for easy, accurate, and reliable measurements of chemotaxis. The method requires only one drop of blood, which is important to prevent anemia in patients with severe injuries. This group of researchers showed that within 24 h, thermal trauma led to a significant decrease in the rate of directed migration, which reached a maximum of 72–120 h after burn injury. Later, Jones et al. [69] described a new phenotype of spontaneous migration of isolated neutrophils in straight microfluidic channels, which made it possible to predict sepsis in patients with severe burns with 80% sensitivity and 77% specificity. This phenotype was observed 1–2 days before the clinical diagnosis of sepsis was made; the test was negative in patients who did not develop sepsis [69, 70].

Neutrophilic migration is suppressed by various inflammatory mediators, which include lipoxins, cytokines (IL-10), and gaseous molecules [71]. Among the gaseous mediators, nitric oxide plays a prominent role in neutrophil migration. Pharmacological inhibition of NOS or a deficiency of the NOS gene was shown to increase the migration of neutrophils to the inflammatory site in response to several stimuli. Currently, the mechanisms by which NO attenuates the neutrophil migration are not well understood. There is evidence that NO produced by eNOS or iNOS modulates the interaction of leukocytes and endothelial cells. Selective inhibitors of iNOS and eNOS increase the adhesion of neutrophils to endothelial cells, while NO donors reduce both the adhesion and transfer of leukocytes to inflammatory sites. In addition, expression of cell adhesion molecules, such as integrins, L-selectin, P-selectin, E-selectin, and ICAM-1, is suppressed by NO donors and controlled by NOS inhibitors [60].

Nitric oxide and its iNOS derivative inhibit neutrophil migration mainly by the following three mechanisms:

1. iNOS inhibits β-integrins and selectins in leukocytes, and also reduces the expression of VCAM-1;
2. NO interacts with other molecules, such as ROS, forming peroxynitrite, which can reduce the chemotactic activity of neutrophils and their interaction with the endothelium, relaying to P-selectin;
3. NO is able to induce the expression of heme oxygenase-1, which may impair the rolling and adhesion of neutrophils.

In mice, anti-inflammatory acute-phase proteins (C-reactive protein, serum amyloid A, alpha-1-acid glycoprotein, pentaxin-3, and hemopexin) suppress migration/chemotaxis of neutrophils [72]. According to the authors, therapeutic inhibition of acute phase proteins can improve neutrophil migration and, as a result, increase survival of septic patients.

Although neutrophils are activated during SIRS, their sensitivity to the fMLP chemotactic stimulus is reduced. This is illustrated by a decrease in the expression of active FcγRII (Fc gamma receptor II) and CD32 on neutrophils. The low functionality of this Fcγ receptor on neutrophils may be associated with the production of immature neutrophils [73]. As shown earlier [38], immature neutrophils under-express antibacterial receptors, such as CD14 and MD-2 (myeloid differentiation factor 2), and are, therefore, less able to transmigrate.

In addition, NSPs released upon degranulation can mediate proteolytic cleavage of receptors on immune cells [74]. Neutrophilic proteases can also target complement receptors. A decrease in the levels of CR1/CD35 and C5aR/CD88 during inflammation was reported [75]; this could impair the interaction of neutrophils with microorganisms.

Thus, the mechanisms that control the chemotactic function of neutrophils in sepsis are complex. The totality of the data suggests that overproduction of cytokines, chemokines, and NO observed during lethal bacterial sepsis is a major factor behind the disruption of neutrophil migration into the infected area.

There is evidence that, in addition to direct antimicrobial function, neutrophils can modulate the adaptive immune responses to severe inflammation [76, 77]. It has been shown that acute inflammation, including burn injury and sepsis, is paralleled by the appearance of nontypical neutrophils in the blood [78, 79].

In 2012, J. Pillay et al. [80] used flow cytometry to observe the appearance of different subtypes of neutrophils in the peripheral blood during acute systemic inflammation caused by the administration of LPS (2 ng/kg) to volunteers. This study was based on the measurement of CD16 (FcγRIII) and CD62L (L-selectin). The authors were able to differentiate between three varieties of “inflammatory” neutrophils: neutrophils with a regular segmented nucleus (CD16bright/CD62Lbright), neutrophils with a ribbon-like nucleus (CD16dim/CD62Lbright) and neutrophils with a hyper-segmented nucleus (CD16bright/CD62Ldim). However, very little is known about the origin of the CD62Ldim cells. It is believed that an increase in nuclear segmentation occurs with an increase in cell age, which is not confirmed by experimental data [78]. Studies using proteomic and kinetic profiling of neutrophils *in vivo* following LPS infection have shown that hyper-segmented neutrophils have the same age as normal segmented cells and the same time to reach maturity; therefore, those cannot be considered senescent cells [78, 80]. Thus, the researchers concluded that the hyper-segmented CD62Ldim cells do not originate from mature neutrophils, but can result from a different process [80]. These cells enter the bloodstream only during inflammation as a separate subset of neutrophils. It was found that CD62Ldim neutrophils had immunosuppressive properties and were able to inhibit T cell proliferation using a ROS-dependent mechanism in the immunological synapse [80, 81].

Another mechanism by which hyper-segmented neutrophils can inhibit T cell responses is the expression of the surface protein PD-L1 (programmed death ligand 1) [82]. INF-γ induces PD-L1 expression by neutrophils, which allows them to suppress cell proliferation and induce lymphocyte apoptosis [34]. The PD-1/PD-L1 axis is believed to be an important mechanism of immune suppression in septic patients. Blocking this axis by a PD-1-blocking antibody improved survival of mice with sepsis [83]. Based on these studies, it was concluded that the PD-1/PD-L1 pathway might become a new therapeutic target in the treatment of sepsis; clinical trials to confirm this hypothesis are yet to be conducted. Such a suppressive mechanism may be protective in tissues with severe inflammatory infiltrates. On the other hand, this approach may turn counterproductive when neutrophils migrate to the lymph nodes and interact with cells of adaptive immunity, as shown in experiments on mice [84, 85].

In addition to CD62Ldim, myeloid-derived suppressor cells (MDSC), that appear in pathological conditions, such as severe burn injury, sepsis, or tumor, also possess immunosuppressive activity [24, 86]. The MDSC population consists of monocyte and granulocyte subpopulations. The mechanism by which MDSCs can suppress T cells involves the expression and secretion of arginase-1, which reduces the concentration of arginine in the microenvironment. L-arginine deficiency leads to the arrest of the T cells cycle at the G0–G1 phase [87].

Thus, severe inflammation and sepsis involve numerous overlapping immunosuppression mechanisms, affecting both innate and adaptive immunity. The present knowledge about the heterogeneity of neutrophils highlights the importance of PMN phenotype its correlation with thermal damage and clinical outcome.

## Monocytes/macrophages

The mononuclear phagocyte system is a critical component of the innate immune response and is involved not only in the recognition and elimination of various microorganisms, but also in the modulation of innate immune responses through the production of pro-and anti-inflammatory cytokines [88, 89].

Available data indicate that the diverse biological activity of macrophages is mediated by phenotypically different subpopulations of cells produced in response to local inflammation [90]. In this aspect, two main cell populations are important: classically activated M1 macrophages and alternatively activated M2 macrophages [91, 92]. Macrophages M1 are activated by: 1) cytokines, such as IFN-γ and TNF-α; 2) pathogen-associated molecular patterns (PAMPs); and 3) endogenous signals of “danger” (for example, heat shock proteins or high-mobility group 1 protein — HMGB1). These cells exhibit potent antimicrobial activity and release IL-12 and IL-23 interleukins, stimulating Th1’s strong pro-inflammatory immune responses. In addition, they have anti-proliferative and cytotoxic activity mediated by ROS, reactive nitrogen species, and pro-inflammatory cytokines (e.g., TNF-α, IL-1, IL-6). It is believed that the M1 population contributes to macrophage-induced tissue damage [92, 93].

The activity of M1 macrophages is balanced by M2 macrophages, which are mainly involved in suppressing inflammation and initiating wound healing [94]. This is achieved by releasing anti-inflammatory cytokines such as IL-4, IL-10, and IL-13. Macrophages M2 also contribute to the resolution of inflammation by removing apoptotic neutrophils (phagocytosis) and by producing mediators essential for tissue remodeling and angiogenesis: those include transforming growth factor (TGF-β), vascular endothelial growth factor (VEGF), and epidermal growth factor (EGF). Macrophages M2 support Th2-associated effector functions and play a key role in the regulation of T cell activity. Based on their diverse functions, the alternatively activated M2 macrophages are further divided into subpopulations called M2a (activated by IL-4 and IL-13), M2b (activated by immune complexes in combination with IL-1β or LPS) and M2c (activated by IL-10, TGF-β, or glucocorticoids) [95–98].

It should be noted that the classification of macrophages into two polarized states simplifies the complex functional characterization of these cells [99]. Activation of macrophages is a dynamic process: the same cells may initially participate in pro-inflammatory and cytotoxic reactions, and then in resolving inflammation and healing of wounds. This illustrates the plasticity of macrophages and their ability to modulate their reactions due to the changes in the microenvironment [100, 101].

After thermal damage, a population of hyperactive macrophages with increased production of mediators, such as TNF-α, IL-6, IL-1, was noted. However, during the anti-inflammatory phase or sepsis, macrophage dysfunction is a key component of general immunosuppression after burns [95].

Using ELISA, Kobayashi et al. [102] studied the peripheral blood in heavily burned patients to determine the production of cytokines by macrophages of various phenotypes. Peripheral blood samples were obtained within two days after admission to the hospital, which corresponded to 1–4 days after the burn injury. The authors analyzed the culture fluids for the presence of IL-10, IL-12, CCL1 (a biomarker of M2b monocytes), CCL17 (a biomarker of M2a monocytes), CXCL13 (a biomarker of M2c monocytes), and CCL2 (a biomarker of neutrophils).

At the baseline, peripheral blood monocytes did not produce IL-12 either with or without stimulation by the staphylococcal antigen; in contrast, IL-10 was found in all monocyte cultures of burn patients (but not in monocytes of healthy controls). After stimulation with the staphylococcal antigen, IL-12 was produced by all monocytes isolated from healthy subjects. This, according to the authors, indicates that severely burned patients carry M2 monocytes. In addition, the enzyme arginase was found in lysates of monocytes of burn patients but not in cell lysates of controls. The result confirms the earlier observation that M2 monocytes produce arginase [103].

At the next stage of the study, it was found that majority of monocytes from the M2 population represented M2b subpopulation [102]. The authors suggested that CCL2, constantly present in the serum of burn patients, was produced by burn-associated neutrophils; the CCL2 is known to stimulate the conversion of resident monocytes into M2b monocytes.

Macrophages of the M2b subtype have poor plasticity and remained in severely burned patients for a long time. In their presence, the patient’s antibacterial protection is significantly suppressed. Therefore, individuals carrying M2b macrophages are more susceptible to various opportunistic infections [104], as shown in mice burned up to 25% of the body surface area [105].

Thus, M2b macrophages can serve a suitable therapeutic target for controlling opportunistic infections in patients with burn injury; this notion though is yet to be proved in further studies.

## Conclusion

Sepsis and septic shock are emergency conditions that often occur in the treatment of burn patients. The diagnosis of sepsis after a severe burn injury is complicated by the overlapping signs of sepsis and systemic inflammatory response. Under these conditions, it is important to clinically identify the patients who are developing an infection in order to start timely antibiotic therapy.

Currently, an extensive research into nonspecific resistance parameters is under way in the hope to predict and/or diagnose life-threatening complications in burn patients. This is one of the promising venues in modern clinical combustiology.
